# Does the reliability of computational models truly improve with hierarchical modeling? Some recommendations and considerations for the assessment of model parameter reliability: Reliability of computational model parameters

**DOI:** 10.3758/s13423-024-02490-8

**Published:** 2024-05-08

**Authors:** Kentaro Katahira, Takeyuki Oba, Asako Toyama

**Affiliations:** 1 Human Informatics and Interaction Research Institute, National Institute of Advanced Industrial Science and Technology (AIST), Central 6, 1-1-1 Higashi, Tsukuba, 305-8566 Ibaraki Japan; 2 Department of Cognitive and Psychological Sciences, Graduate School of Informatics, Nagoya University, Nagoya, Japan; 3 Japan Society for the Promotion of Science, Tokyo, Japan; 4 Graduate School of the Humanities, Senshu University, Kawasaki, Japan; 5 Graduate School of Social Data Science, Hitotsubashi University, Tokyo, Japan

**Keywords:** Computational modeling, Parameter estimation, Reliability, Hierarchical modeling, Heterogeneity, Intersession variability, Reinforcement learning models

## Abstract

Computational modeling of behavior is increasingly being adopted as a standard methodology in psychology, cognitive neuroscience, and computational psychiatry. This approach involves estimating parameters in a computational (or cognitive) model that represents the computational processes of the underlying behavior. In this approach, the reliability of the parameter estimates is an important issue. The use of hierarchical (Bayesian) approaches, which place a prior on each model parameter of the individual participants, is thought to improve the reliability of the parameters. However, the characteristics of reliability in parameter estimates, especially when individual-level priors are assumed, as in hierarchical models, have not yet been fully discussed. Furthermore, the suitability of different reliability measures for assessing parameter reliability is not thoroughly understood. In this study, we conduct a systematic examination of these issues through theoretical analysis and numerical simulations, focusing specifically on reinforcement learning models. We note that the heterogeneity in the estimation precision of individual parameters, particularly with priors, can skew reliability measures toward individuals with higher precision. We further note that there are two factors that reduce reliability, namely estimation error and intersession variation in the true parameters, and we discuss how to evaluate these factors separately. Based on the considerations of this study, we present several recommendations and cautions for assessing the reliability of the model parameters.

**Supplementary Information:**

The online version contains supplementary material available at 10.3758/s13423-024-02490-8.

## Introduction

Computational modeling of behavioral and neural data is becoming a standard methodology in psychology, cognitive neuroscience, and computational psychiatry (Daw, 2011; Maia & Frank, 2011; Farrell & Lewandowsky, 2018). This approach often involves fitting computational (or cognitive) models, such as reinforcement learning models, to behavioral data acquired from cognitive tasks. In computational psychiatry, the relationship between model parameter estimates and psychiatric disorders has been explored (Huys, Pizzagalli, Bogdan, & Dayan, 2013; Huys, Maia, & Frank, 2016; Robinson & Chase, 2017). Such computational modeling is expected to enable the realization of a ‘computational assay’ that can infer patient-specific disease processes from behavioral or neural data (Stephan & Mathys, 2014; Browning et al., 2020). For instance, it is expected that these parameter estimates (also termed *computational measures*) can be used to evaluate how therapeutic interventions, such as pharmacological treatments or psychotherapy, influence cognitive functions and behaviors in patients (Wheaton, Gillan, & Simpson, 2019; Yip et al., 2022; Hauser, Skvortsova, De Choudhury, & Koutsouleris, 2022).

Importantly, the parameter estimates, particularly point estimates, inevitably contain an estimation error or uncertainty.^1^ A larger magnitude of this error or uncertainty reduces the likelihood of accurately identifying a true association between behavioral characteristics and psychiatric disorders (Zorowitz & Niv, 2023). One metric that quantifies the error of point estimates is reliability. Reliability indicates the consistency between estimates obtained when the same measurements (data acquisition and parameter estimation procedures) are repeated for the same individual (a human participant or animal subject; Browning et al., 2020; Karvelis, Paulus, & Diaconescu, 2023; Zorowitz & Niv, 2023). The reliability of parameters in computational models has received particular attention in recent years, especially in computational psychiatry (Brown, Chen, Gillan, & Price, 2020; Haines, Sullivan-Toole, & Olino, 2023; Mkrtchian, Valton, & Roiser, 2023). Reliability is directly related to the strength of the correlation between the parameter estimates and other external variables (e.g., self-reported symptom scores or neural activity) and thus to the probability of detecting significant correlations (Haines et al., 2023; Zorowitz & Niv, 2023; see Appendix A for mathematical details). Thus, methods to obtain reliable parameter estimates from cognitive tasks are essential for the realization of effective computational assays (Zorowitz & Niv, 2023; Karvelis et al., 2023).

**Fig. 1:**
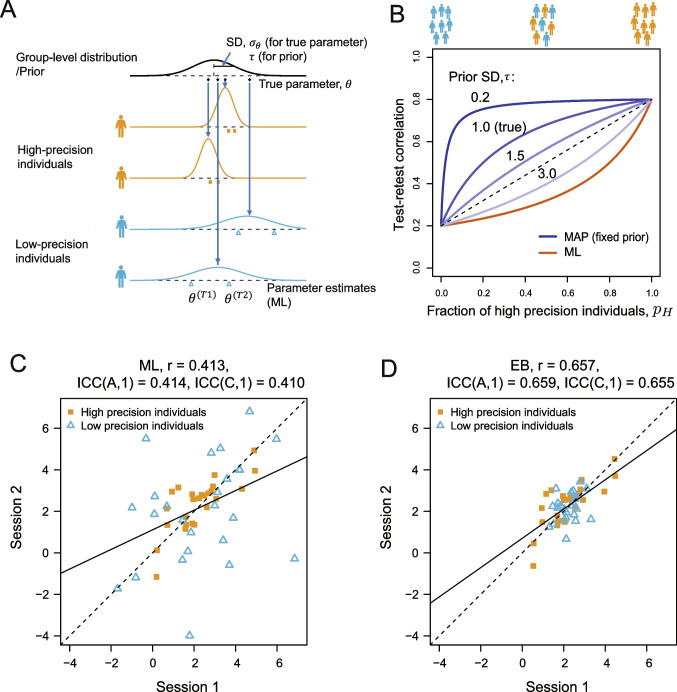
Illustration of the test–retest correlation of parameter estimates. **A** Schematic of the data generation process. The true parameters for each individual are assumed to be drawn from a group-level distribution (here, a Gaussian distribution; the *black bold line*). The parameters for each individual are estimated (denoted as $\hat{\theta }^{(T1)}$, $\hat{\theta }^{(T2)}$) from the data observed in two task sessions. **B** Theoretical value of the test–retest correlation in Gaussian response models as a function of the proportion of high-precision individuals. The *blue lines* show the test–retest correlation of the MAP estimates for varying SDs of the prior. The *red line* indicates that for the estimates of the ML estimation. **C**, **D** Test–retest correlation of the parameter estimates in the first session (T1) and in the second session (T2). *Dotted lines* represent lines of equality, while the *solid lines* indicate regression lines. **C** Scatterplot of point estimates obtained by ML estimation. **D** Scatterplot of point estimates obtained by empirical Bayes (EB). *MAP*, maximum a posteriori; *ML*, maximum likelihood; *r*, Pearson’s correlation coefficient; *ICC(A,1)*, agreement intraclass correlation; *ICC(C,1)*, consistency intraclass correlation

To improve the reliability of computational models, previous research has focused on improving not only behavioral measurements (i.e., cognitive tasks) but also parameter estimation methods (Brown et al., 2020; Waltmann, Schlagenhauf, & Deserno, 2022; Karvelis et al., 2023; Zorowitz & Niv, 2023; Haines et al., 2023). Several researchers have reported that hierarchical Bayesian methods significantly improve the reliability of parameter estimates, compared to that of the case when conventional maximum likelihood (ML) estimation is independently applied to each individual (Brown et al., 2020; Waltmann et al., 2022). Hierarchical modeling approaches assume prior distributions or group-level distributions from which individual parameters are assumed to be generated (Rouder & Lu, 2005; Scheibehenne & Pachur, 2015; see Fig. 1A). These distributions, simply called *priors*, can also be estimated from data via empirical Bayes (EB) methods or by hierarchical Bayesian approaches. Estimates of individual-level parameters are often obtained by maximum a posteriori (MAP) estimation, whether the priors are fixed a priori or determined by EB. The MAP estimates for each individual are stabilized by constraining the estimates with priors (Ahn, Krawitz, Kim, Busemeyer, & Brown, 2011; Katahira, 2016; Gershman, 2016). In this paper, unless otherwise noted, we refer to the estimates when the prior is fixed a priori as *MAP estimates* and the estimates when the prior is determined by the EB as *EB estimates*.

The use of priors can lead to greater correlations between point estimates and external variables when there is heterogeneity in estimation precision within the population (Katahira, 2016). Such heterogeneity can arise, for example, when the number of trials varies across individuals (with larger trials leading to higher precision) or when there is a mixture of individuals with high and low variability in responses. Furthermore, as we show, the precision of estimates can vary depending on the true values of each parameter. An improvement in reliability due to priors is likely to occur in such situations. This is because reliability measures such as test–retest reliability also reflect correlations between parameter estimates from different sessions, and theoretically, the same theory applies to correlations with an external variable. If this is true, do priors truly improve reliability in a meaningful sense? What implications do various reliability assessment metrics have for these scenarios? We discuss these questions through theoretical analysis and numerical simulations.

Another point discussed in this review of the computational model parameters is that factors that reduce the reliability of parameters can be decomposed into at least two meaningful components: First, the factors that cause the estimates to deviate from the true values of the parameters in the estimation procedure are calculated. Second, the factor that causes the true parameters to vary from session to session (*intersession variability*) due to the individuals’ state, such as mood (Karvelis et al., 2023; Palminteri & Chevallier, 2018).^2^ However, researchers evaluating parameter reliability have rarely explicitly distinguished between these factors. It is important to distinguish between these two factors to determine ways to improve parameter reliability. Estimation errors can potentially be mitigated through refinement of estimation methods, alongside improvements in task design. However, the influence of intersession variation in the true parameter is best addressed through modifications in task design and experimental procedures, rather than through estimation methods. We discuss how the degree of attenuation in reliability due to intersession variability can be estimated separately from the influence of estimation error.

Recently, a method has been proposed to jointly model the population data of two sessions and to derive the parameter reliability from the covariance matrix of the group-level distribution (Brown et al., 2020; Waltmann et al., 2022; Sullivan-Toole, Haines, Dale, & Olino, 2022; Mkrtchian et al., 2023). The reliability obtained in this way tends to be greater than that assessed by classical methods, suggesting that this joint modeling approach enhances reliability. However, such derived reliability may primarily reflect the intersession correlation of the true parameter rather than the effect of estimation error. Consequently, this reliability measure may only measure some of the factors that decrease reliability rather than enhance it.

This paper is organized as follows. First, standard measures of reliability based on classical test theory are reviewed. Then, we describe how the parameter estimates of the computational model deviate from those of classical test theory, especially when there is heterogeneity in estimation precision, and we discuss how these deviations affect the reliability measures. As a specific example, we consider a Gaussian response model. This model has the advantage that the true values of a reliability measure can be obtained analytically and that its underlying principles are easy to interpret. Next, we consider a more practical example of computational modeling of behavioral data, in which a reinforcement learning model is fitted to choice data in a probabilistic reversal learning task. We then discuss the effect of intersession variation and how to estimate the degree of reliability loss due to intersession variation separately from the effect of estimation error. Finally, based on the issues discussed in this paper, we provide recommendations for evaluating the reliability of the model parameters.

## Reliability and test–retest correlation

In general, reliability refers to the degree to which similar observations (or scores) are obtained when similar measurements are made. In parameter estimation, measurement corresponds to the administration of a cognitive task and the estimation of parameters from the data, whereas observation corresponds to the parameter estimates. Reliability is a metric for the precision of a measurement and is distinguished from validity, which is the degree to which the quantity to be measured is truly being measured. There are several protocols for assessing reliability (see Haines et al., 2023). Among these protocols, test–retest reliability is commonly used to evaluate the reliability of the parameters of a computational model. Test–retest reliability is a measure that quantifies the degree to which a similar observed value is consistently observed when the same individual performs the same task multiple times. Typically, the same individual is asked to perform the same task several days to several months apart, and the degree of consistency between the parameter estimates is evaluated. The various forms of the intraclass correlation coefficient (ICC) (Shrout & Fleiss, 1979; Koo & Li, 2016; McGraw & Wong, 1996) and the Pearson’s correlation coefficient are often used to evaluate the degree of consistency.

In classical test theory, reliability is formally defined as the ratio of the variance of the parameter of interest to the total observed variance. Specifically, reliability is expressed as

1 $$\begin{aligned} \text {Reliability}&= \frac{\text {Variance of interest}}{\text {Total variance}} \nonumber \\&= \frac{\text {Variance of interest}}{\text {Variance of interest} + \text {Unwanted variance}}. \end{aligned}$$

### Case with a constant true parameter

First, let us consider a simple scenario in which the true parameter does not vary across sessions. That is, the parameter is fixed at a single point for each individual and does not change over time. In this case, the ‘variance of interest’ is the variance of the true parameter across individuals. The ‘unwanted variance’ can be taken as the variance of the estimation error per session. We denote this error variance by $\sigma _\epsilon ^2$, and we assume that $\sigma _\epsilon ^2$ is common within the population. The variance of the true parameter, $\theta$, across individuals is denoted by $\sigma _{\theta }^2$. Given this assumption, the reliability of the parameter estimator can be formulated as follows:

2 $$\begin{aligned} \text {Reliability} = \frac{\sigma _{\theta }^2}{\sigma _{\theta }^2 + \sigma _{\epsilon }^2}. \end{aligned}$$

This quantity is evaluated using ICCs.

There are two types of ICCs that are often used to evaluate the test–retest reliability of parameter estimates. *Agreement ICC* or ICC(A,1) (in the notation used by McGraw and Wong (1996)) provides an absolute agreement or agreement estimate between measurements (i.e., parameter estimates) and does not allow for any systematic errors. *Consistency ICC* or ICC(C,1) allows for systematic offsetting errors (but not scaling factor errors) between measurements. For details on their calculations, readers are referred to Liljequist, Elfving, and Skavberg Roaldsen (2019).

Under certain conditions, the Pearson correlation coefficient calculated between parameter estimates derived from two separate sessions can also serve as an estimate of this quantity. We refer to this coefficient as the “test–retest correlation” following Scheibehenne and Pachur (2015), rather than “test–retest reliability,” as we investigate scenarios where test–retest correlation may not be an appropriate measure of reliability. Although the distinctions between ICCs and test–retest correlation coefficients are explored later, in many contexts considered in this study, these metrics are generally consistent. The following discussion is mainly focused on the test–retest correlation.

Test–retest correlation and ICCs can be appropriate measures of reliability in terms of classical test theory under the following conditions (see Appendix B): (i) The variance of the error of the parameter estimates (or its square root, the standard error [SE]) is the same across individuals (homogeneity assumption); (ii) The estimation errors for the first and second measurements (sessions) are independent. We discuss what happens to the test–retest correlation obtained by using priors when these assumptions are not met. A typical situation that violates the first condition is when there is heterogeneity in estimation precision, with a mixture of individuals who are inattentive to the task and those who are not (Zorowitz, Solis, Niv, & Bennett, 2023). The second condition, independence between sessions, may not be satisfied if the hierarchical model is jointly estimated across two sessions.

## Influence of heterogeneity in estimation precision

### Theoretical considerations based on the Gaussian response model

Here, we examine the effect of heterogeneity in the estimation precision on the test–retest correlation by using a Gaussian response model (Gelman et al., 2013; Katahira, 2016). With this model, the population value (true value) of the test–retest correlation can be obtained analytically (without numerical simulation), which facilitates theoretical interpretation (see Appendix C for details). Throughout this paper, to emphasize the impact of heterogeneity in estimation precision, we assume two distinct groups: individuals with clearly low estimation precision and those with clearly high estimation precision, referred to as low-precision individuals and high-precision individuals, respectively. Let $p_H$ be the proportion of high-precision individuals in the population. We vary $p_H$ and see how this affects the test–retest correlation. Here, we assume a Gaussian prior (group-level distribution) for the individual mean response parameter, $\theta _i$ (the bold black line in Fig. 1A). The distribution of parameter estimates for each individual (the thin colored lines in Fig. 1A) depends on the variance of the response. The inverse of the variance of the distribution corresponds to the precision (i.e., a smaller variance corresponds to higher precision). Individuals with low precision (e.g., those who are inattentive to the task) correspond to those with a broader distribution (higher variance and thus lower precision). Here, we assume that the true parameters do not change between test–retest sessions. Thus, if there is no estimation error, the test–retest correlation will be one.

Figure 1C shows an example of a test–retest correlation when ML estimation (without priors) is applied in the presence of such heterogeneity, generated by a simulation with $p_H = 0.5$. This method yields a relatively low test–retest correlation ($r = 0.413$), mainly due to the influence of the low-precision individuals (blue triangles). Figure 1D shows the result of MAP estimates obtained with the EB, where the parameters of the prior are adjusted according to the data from the entire population (see Appendix C). In this case, the test–retest correlation is relatively high ($r = 0.657$). This is because the estimates of the low-precision individuals move toward the prior mean and have a lower influence on the correlation. Figure 1C and D also show the values of the two types of ICCs, which in this case are almost the same as the correlation coefficient.

Figure 1B shows the theoretical values of the test–retest correlation for this model as a function of the proportion of high-precision individuals, $p_H$ (the analytical expression of the function is given in Eqs. 20 and 21 in Appendix C). Here, the true standard deviation (SD) of the distribution of $\theta$ is set to $\sigma _{\theta } = 1$, the SE ($\sigma _{\epsilon }$) of the low-precision individuals is 2.0, and the SE of the high-precision individuals is 0.5. If there are only low-precision individuals ($p_H = 0$), we find that the true population test–retest correlation (and also the ICC) is $1/(1+2^2) = 0.2$ by substituting $\sigma _{\theta } = 1$ and $\sigma _{\epsilon } = 2$ into Eq. 2. When there are only high-precision individuals ($p_H = 1$), the test–retest correlation is $1/(1+0.5^2) = 0.8$. Note that the prior (regardless of the SD) does not affect the test–retest correlation in these situations (this can be confirmed by substituting $p_H = 0$ or $p_H = 1$ in Eq. 21).

When both types of individuals are present in a population, i.e., when there is heterogeneity in the estimation precision, the prior influences the test–retest correlation. The theoretical value of the test–retest correlation when using the ML (without prior data) is $1/(1+((1-p_H) \times 2^2 + p_H \times 0.5^2))$ (the red line in Fig. 1B). Note that this value is always lower than the intergroup average of the test–retest correlations (dashed line in the figure; $(1-p_H) \times 0.2 + p_H \times 0.8$). Thus, the test–retest correlation for MLs underestimates the average correlation of a single individual within the group. In fact, this test–retest correlation is the same as the reliability of a population where every individual has an estimation error variance that equals the mean error variance across individuals (that is, $(1-p_H) \times 2^2 + p_H \times 0.5^2$). However, since this term appears in the denominator of the definition of reliability (Eq. 2, i.e., it acts as a divisor), the test–retest correlation is a nonlinear function of $p_H$.

The test–retest correlation of MAP estimation depends on the SD of the prior, denoted by $\tau$, as shown by the blue lines in Fig. 1B. When $\tau$ coincides with the true value of the group-level distribution ($\tau = \sigma _{\theta } = 1.0$), the result of its test–retest correlation is greater than the intergroup average test–retest correlation (dashed line). Furthermore, if $\tau$ is less than the true value (i.e., $\tau = 0.2$), the result of the test–retest correlation primarily reflects the reliability of high-precision individuals, even if their proportion is small. Thus, the presence of low-precision individuals can be largely ignored. Conversely, as the SD of the prior $\tau$ increases, the effect of the prior distribution decreases, and the test–retest correlation approaches that of the ML estimation (red line). See Appendix C for a theoretical explanation of the effects of the prior variances. Notably, the tendency to give more weight to samples with higher precision in test–retest correlations can be observed to some extent even when the variance of the prior is equal to the true group-level distribution (i.e., $\sigma _\theta = \tau$), that is, when no extreme constraints are imposed.

**Fig. 2:**
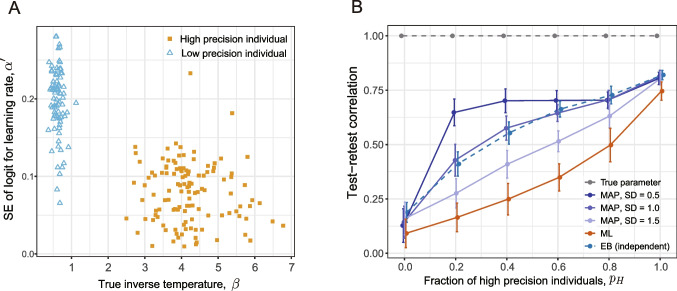
The effects of heterogeneity in estimation precision of parameters of a reinforcement learning model. The scenario where the true parameter remains constant across sessions (Case 1). **A** Standard error as a function of true inverse temperature. MAP estimates from a single simulation run ($p_H = 0.4$, SD($\alpha ^\prime$) = 1) were used. **B** Test–retest reliability of estimates for learning rate, $\alpha$, as a function of the proportion of individuals with high precision. The proportion of high precision individuals (with a large inverse temperature parameter, $\beta$) varied in 0.2 increments from 0 to 1. For each proportion, parameter estimation was performed by using MAP, ML, and EB. For MAP, the SD of the logit for the learning rate, $\alpha ^\prime$, was varied between 0.5, 1.0, and 1.5. For each condition and estimation method, the simulations were run 50 times, except for EB, where the results of only ten simulations were used due to computational time constraints. The mean of the test–retest correlation was plotted. *Error bars* represent $\pm 1$ SD. *MAP*, maximum a posteriori; *ML*, maximum likelihood; *EB*, empirical Bayes

### Reinforcement learning model

Next, we investigate whether the effects of prior distributions observed in the Gaussian response model are observed in more realistic computational model fitting. Specifically, we consider an example of fitting a reinforcement learning model in a reward learning task (stochastic reward reversal task) (e.g., Waltmann et al., 2022). In this task, two options (‘A’ and ‘B’) are presented in each trial, and the individual chooses one of them. The outcome (reward or absence of reward) is provided with a probability depending on the choice (see Appendix D.2 for details). The standard reinforcement learning model considered here has two parameters at the individual level: the learning rate, denoted as $\alpha$, and the inverse temperature, $\beta$. For options A and B, the model represents the action value on trial *t* as $Q_t(A)$ and $Q_t(B)$, respectively, which are estimates of the expected reward value.

When an option $c_t$ is chosen on trial *t*, an outcome $R_t$ is obtained ($R_t = 1$ if there is a reward: $R_t = 0$ if there is no reward), the action value (expected reward) is updated according to

3 $$\begin{aligned} Q_{t+1}(c_t) = Q_t(c_t) + \alpha (R_t - Q_t(c_t)). \end{aligned}$$

Here, $0 \le \alpha \le 1$ denotes the learning rate, which controls how much to update from a single outcome. For the unchosen option, the action value does not change.

The choice probabilities are determined by a softmax function with the inverse temperature $\beta$:

4 $$\begin{aligned} P(c_t = A) = \frac{1}{1 + \exp (-\beta (Q_t(A) - Q_t(B)))}, \end{aligned}$$

The larger the inverse temperature $\beta$ is, the more deterministic the choice becomes, and the more likely the model is to choose the more valuable option. The smaller $\beta$ is, the more random the choice becomes.

Let us consider the situation where the learning rate, $\alpha$, is the parameter of interest and there are two groups for estimation precision regarding $\alpha$. This precision is influenced by the inverse temperature parameter $\beta$. A low $\beta$, indicating higher choice randomness, results in poorer estimation precision for $\alpha$, as value updates may not significantly influence the choice. Conversely, a high $\beta$ enhances the estimation precision of $\alpha$, as action value updates are more likely to be reflected in the choice sequence. Figure 2A plots the SE of the estimation error calculated in the simulation as a function of true $\beta$. The graph indicates lower estimation precision (higher SE) for individuals with smaller $\beta$ values. Low $\beta$ individuals are termed ‘low precision’ individuals, whereas high $\beta$ individuals are termed ‘high precision’ individuals. Individuals with a low $\beta$ in the context of reinforcement learning models can be considered models of inattentive individuals (Zorowitz et al., 2023). In fact, Zorowitz et al. (2023) reported that participants suspected of exhibiting careless/insufficient effort (C/IE) responses in their online study, who composed approximately 20% of the total participants, demonstrated significantly lower $\beta$ values compared to those not showing C/IE responses. Furthermore, it has been generally observed that patients with psychiatric disorders exhibit lower values in terms of the parameters of reinforcement learning models, such as the learning rate and inverse temperature (Robinson & Chase, 2017; Pike & Robinson, 2022). This finding suggests that the estimation precision may be lower in these patients.

We simulated the probabilistic reversal learning task for two sessions by using a model with the same parameters for each individual, varying the proportion of high-precision individuals (those with higher $\beta$), $p_H$, and observed how the test–retest correlation of the estimates changed (see Appendix D.2 for details). For these data, the parameters were estimated with ML, MAP with a fixed prior, and EB. For the MAP, the simulation was performed while varying the variance of the prior for $\alpha$. Examples of parameter estimates for each estimation method are shown in Supplementary Figs. S1 and S2B.

The resulting test–retest correlation for $\alpha$ is shown in Fig. 2B. Similar trends as those in the Gaussian response model were observed: When the whole population is a low-precision individual ($p_H = 0$) or when the whole population is a high-precision individual ($p_H=1$), the effect of the prior is small, and the test–retest correlation is similar between the different estimation methods.^3^ When there is heterogeneity in the estimation precision (i.e., $0.2 \le p_H \le 0.8$), the effect of the prior becomes more pronounced: In MAP with a fixed prior, as the variance of the prior decreases (indicating a stricter constraint), the test–retest correlation approaches the value observed when $p_H = 1$. Even when the variance of the prior equals that of the true group-level distribution (SD = 1.0), the test–retest correlation was slightly biased toward high-precision individuals; that is, the test–retest correlation is greater than the line connecting the results for $p_H = 0$ and $p_H=1$.

The results of the EB estimates (Fig. 2B, blue broken line) are close to the MAP estimation results when the variance of the true prior is equal to the group-level distribution (SD = 1). That is, even with the prior estimate of EB, the test–retest correlation is still biased toward high-precision individuals.

In this study, we focused on the heterogeneity of the estimation precision rather than the heterogeneity of the parameter values themselves. For the inverse temperature $\beta$, the estimation precision is greater for high-precision individuals with high $\beta$, as is the case for $\alpha$. However, unlike $\alpha$, there is more heterogeneity in the value itself between groups. Therefore, the test–retest correlation is greater in heterogeneous cases (see Supplementary Fig. S2) because the variance in the true parameter value (corresponding to $\sigma _\theta ^2$ in Eq. 2) is also greater in these cases.

**Fig. 3:**
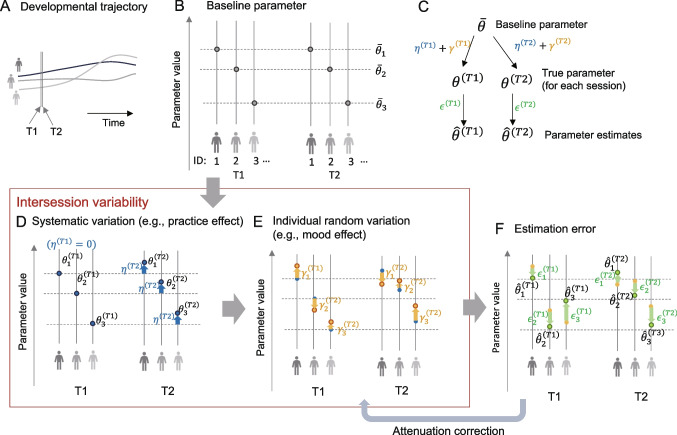
Schematic of how true parameter varies and parameter estimates are obtained. **A** The slowest temporal changes associated with development and aging. **B** The baseline parameter $\bar{\theta }$, reflecting the individual’s traits, is assumed to be constant within individual during test–retest interval (T1, T2). **C** The mathematical representation of the schematic model. **D**, **E** Intersession variability. The intersession variability added to the baseline parameter includes systematic variation common to the population (**D**) and random variation that differs across individuals (**E**). The parameter estimates for each session are obtained after estimation error is added (F)

### Interim discussion

While hierarchical modeling is believed to improve the test–retest reliability of parameter estimates in computational models, we note that such a situation occurs only when there is heterogeneity in the variance of the estimation error. Such heterogeneity departs from classical test theory, in which it is assumed that error variances are shared across individuals.

However, test–retest correlations and ICCs are still meaningful for a typical correlation analysis, which examines the association between individual parameters and external variables such as symptom scores (e.g., Huys et al., 2012; Harlé, Guo, Zhang, Paulus, & Yu, 2017; Oba, Katahira, & Ohira, 2021). Specifically, they are meaningful indicators for estimating how the correlation between parameter estimates and an external variable is attenuated by estimation error; the square root of the reliability of the parameter estimates is the attenuation factor for the true correlation coefficient (see Appendix A for the mathematical details). This property holds even when there is heterogeneity in the estimation precision.

We note, however, that the test–retest correlation is a meaningful statistic for the population as a whole and not necessarily for all individuals in the population. For example, if there is a mixture of individuals who are attentive to the task and those who are not (a situation that is especially likely to occur in online experiments by Zorowitz et al., 2023), the test–retest correlation may be estimated to be high because the test–retest correlation may be estimated to be greater when the results of attentive individuals are biased by priors. This high reliability does not apply to the precision of parameter estimation for inattentive participants. If such inattentive individuals are patients with a disorder of interest (e.g., ADHD patients), the reliability measure obtained may not be useful for estimating the correlation between symptom severity and model parameters across patients. The extent to which reliability measures obtained in a given study can be generalized must be clarified by the nature of the population being analyzed. If heterogeneity in estimation precision is suspected, it may be necessary to assess reliability by subdividing the population into homogeneous groups.

## Intersession variability of true parameters

Thus far, we have assumed that the true parameter values of the model are constant within individuals during the interval spanning two sessions (test, retest sessions). In reality, however, ‘state-like fluctuations’ due to mood, sleepiness, attentiveness, and practice effects may cause true parameters to vary from session to session (Karvelis et al., 2023; Palminteri & Chevallier, 2018). Such intersession variation in the true parameters should be treated differently from the estimation errors we have considered. Such variation is likely to depend on the task design, the interval between two sessions and the timing of administration, rather than on the estimation method. In this section, we first consider how such a process can be represented in a conceptual model, and then, we discuss how intersession variability affects different reliability measures and how each measure should be selected and treated.

We present a conceptual model that describes the process of generating parameter estimates from true parameters with intersession variation (Fig. 3). Changes in the process of an individual’s behavior can occur over multiple time scales, from changes in life-course changes to microvariations due to learning within a task (Palminteri & Chevallier, 2018). The slowest changes occur for individual traits that change slowly with development, aging, and disease progression (Fig. 3A). The interval between two sessions in which test–retest reliability is examined can be as long as a few months, during which time the traits can be approximated as stable and constant (Fig. 3B). The baseline parameter $\bar{\theta }_i$ for the $\i$th individual is assumed to reflect the traits of this period and does not change between sessions (Fig. 3B). In addition, systematic variations, such as practice effects and circadian effects, are common within the population (Fig. 3D). There are also individual-specific random variations, such as mood effects (Fig. 3E). We denote the former systematic variation by $\eta ^{(t)}$ and the latter individual and session-specific random effects by $\gamma _{i}^{(t)}$, where *t* is the session ID ($t = T1,T2$). We assume that the true parameter for session *t*, $\theta _{i}^{(t)}$, is obtained by adding these session-specific effects to the baseline parameter, i.e., $\theta _{i}^{(t)} = \bar{\theta }_i + \eta ^{(t)} + \gamma _{i}^{(t)}$. The true parameter $\theta _{i}^{(t)}$ plus the estimation error $\epsilon _{i}^{(t)}$ yields the parameter estimate $\hat{\theta }_{i}^{(t)}$ (Fig. 3F).

Systematic variation, $\eta ^{(t)}$, can be treated as a fixed effect if the effect is consistent across studies, as in the practice effect. Alternatively, it can be viewed as a random effect if it is consistent within studies but varies across studies, such as in the case of seasonal effects. Let $\sigma _{\eta }^2$ be the variance of the systematic variation, $\eta ^{(t)}$. The variance of the random variation, $\gamma _i$, which varies from individual to individual, is $\sigma _{\gamma }^2$ (here, it is assumed that the values are common to all individuals). If the variance of interest is the baseline parameter $\bar{\theta }$ and the variance of the estimate is considered to include the systematic variation $\eta ^{(t)}$, then the reliability in this case is defined as

5 $$\begin{aligned} \text {Reliability} = \frac{\sigma _{\bar{\theta }}^2}{\sigma _{\bar{\theta }}^2 + \sigma _{\eta }^2 + \sigma _{\gamma }^2 + \sigma _{\epsilon }^2}. \end{aligned}$$

In such cases, what does the test–retest correlation represent? According to Appendix B.2, assuming that both $\sigma _{\gamma }^2$ and $\sigma _{\epsilon }^2$ are uniform within the population, the population correlation coefficient is given by:

6 $$\begin{aligned} \rho [\hat{\theta }^{(1)}, \hat{\theta }^{(2)}] = \frac{\sigma _{\bar{\theta }}^2}{\sigma _{\bar{\theta }}^2 + \sigma _{\gamma }^2 + \sigma _{\epsilon }^2}. \end{aligned}$$

The variance appearing in the correlation coefficient calculation is the quantity after subtracting the mean in each session. Thus, the effect of $\eta ^{(t)}$ vanishes, and $\sigma _{\eta }^2$ does not appear. The sum $\sigma _{\gamma }^2 + \sigma _{\epsilon }^2$ is considered the error variance.^4^ The ICC(C,1) can also yield the same value if there are no differences in the variance of the estimates across sessions.

On the other hand, ICC(A,1) takes into account the effect of systematic variation between sessions, represented by $\sigma _{\eta }^2$, in the variance of parameter estimates (ICC(A,1) decreases in the presence of bias). In other words, the reliability of Eq. 5 is evaluated. However, in ICC(A,1), as well as in ICC(C,1) and Pearson correlation, the session-specific random variation $\gamma _{i}$ cannot be distinguished from the estimation error $\epsilon _{i}$. Thus, $\sigma _{\gamma }^2 + \sigma _{\epsilon }^2$ are collectively treated as the variance of error.^5^

## Isolating the effects of intersession variation

In this section, we consider a method to evaluate the decrease in reliability due to intersession variability separately from the decrease in reliability due to intrasession estimation errors.

The test–retest correlation decomposes into products of three correlations as follows (proof is given in Appendix B.2):

7 $$\begin{aligned} \rho [\hat{\theta }^{(T1)}, \hat{\theta }^{(T2)}] \!=\! \rho [\theta ^{(T1)}, \theta ^{(T2)}] \cdot \rho [\theta ^{(T1)}, \hat{\theta }^{(T1)}] \cdot \rho [\theta ^{(T2)}, \hat{\theta }^{(T2)}], \end{aligned}$$

where $\rho [\theta ^{(T1)}, \theta ^{(T2)}]$ is the intersession correlation of the true parameters, and $\rho [\theta ^{(T1)}, \hat{\theta }^{(T1)}]$ and $\rho [\theta ^{(T2)}, \hat{\theta }^{(T2)}]$ are the intrasession correlations of the true and estimated parameters in sessions 1 and 2, respectively. If there is no between-session difference in the variance of estimation error, these two can be considered equivalent. Let $\rho [\theta , \hat{\theta }]$ denote that correlation (i.e., $\rho [\theta , \hat{\theta }] = \rho [\theta ^{(T1)}, \hat{\theta }^{(T1)}] = \rho [\theta ^{(T2)}, \hat{\theta }^{(T2)}]$). From the relationship of Eq. 7, for example, if $\rho [\theta ^{(T1)}, \theta ^{(T2)}]$ is known, the degree of decrease in reliability due to estimation error, $\rho [\theta , \hat{\theta }]$, can be inferred from the test–retest correlation, $\rho [\hat{\theta }^{(T1)}, \hat{\theta }^{(T2)}]$. Conversely, if $\rho [\theta , \hat{\theta }]$ is known, the degree of decrease in reliability due to variations in the true parameters, $\rho [\theta ^{(T1)}, \theta ^{(T2)}]$, can be inferred.

In principle, the correlation between the true and the estimated values of a parameter, $\rho [\theta ^{(t)}, \hat{\theta }^{(t)}]$, can be evaluated via parameter recovery analysis (Palminteri, Wyart, & Koechlin, 2017). This involves specifying the true model and the distribution of true values and generating data based on an assumed model. Then, the parameters are estimated from the data, and their correlation with the true parameters is calculated. Recently, this procedure has been recommended for accompanying model fitting (Wilson & Collins, 2019). However, parameter recovery requires determining the distribution of the true parameters, and it is assumed that the true model and the distribution of the true parameters are correct. Next, we show how to estimate the correlation between sessions of the true parameters, $\rho [\theta ^{(T1)}, \theta ^{(T2)}]$, by eliminating the effect of estimation error (i.e., $\rho [\theta ^{(t)}, \hat{\theta }^{(t)}]$).

### Attenuation correction

We present a method for estimating the intersession correlation of true parameters by using individual-level parameter estimation results (A more direct way to incorporate the computational model as an individual-level model is discussed in a later section). This method relies on a framework that corrects for the attenuation of correlations by using information about the variance of the parameter estimation error. In our schematic in Fig. 3, this procedure corresponds to the arrow from panel F to panel E. Specifically, we consider a framework of Bayesian estimation assuming a hierarchical generative model as a flexible method that can be applied even when there is heterogeneity in the estimation precision (Behseta, Berdyyeva, Olson, & Kass, 2009; Matzke et al., 2017; Rouder & Haaf, 2019).

In this framework, we assume that the true parameters at the individual level are generated from a bivariate Gaussian distribution (see Appendix D.3 for details). The observed values (parameter estimates) are supposed to be obtained by adding Gaussian noise to the true parameters. Based on this hierarchical model, the posterior distribution of the correlation coefficients between the true parameters across sessions is calculated from the covariance matrix of the group-level distribution. We refer to this method as Bayesian attenuation correction. If the SE of each observation point is assumed to be zero, the method corresponds to a Bayesian inference for Pearson’s correlation coefficient.

**Fig. 4:**
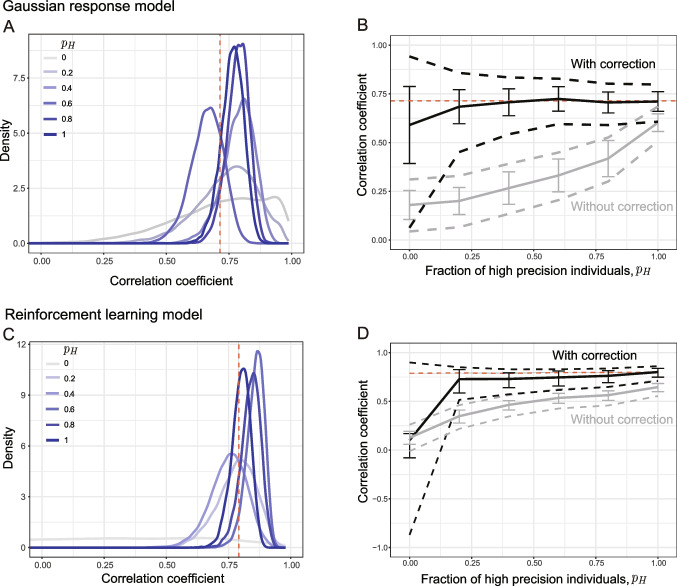
Inference for intersession correlation of the true parameter with Bayesian attenuation correction. **A**, **B** Gaussian response model; **C**, **D** reinforcement learning model. **A**, **C** Examples of posterior distributions of the corrected correlation are plotted for each value of $p_H$. The *dashed vertical red line* represents the true value of the correlation coefficient for the true parameter values. **B**, **D** Mean of the posterior distribution for 50 simulations (*solid lines* represent the mean across simulations, with *error bars* indicating SD) and 95% credible interval (*broken lines* represent the mean across simulations). The *black lines* represent the posterior distribution after Bayesian attenuation correction, and the *gray lines* represent the posterior distribution of correlation coefficients without correction. The *dashed red line* represents the population correlation coefficient for the true parameter values

### Illustration with the Gaussian response model

We apply this Bayesian attenuation correction to the Gaussian response model and to the reinforcement learning model. For the Gaussian response model, the SEs of the individual-level parameters utilized for Bayesian attenuation correction are assumed to be known. In contrast, in the reinforcement learning model, the SEs of the individual-level parameters are not known a priori. Instead, we utilize coarse approximations computed during the parameter estimation process (see Appendix D.3). Consequently, the extent to which the correlation between sessions of true parameter values can be accurately estimated remains uncertain.

The results for the simulated data when using the Gaussian response model are shown in Fig. 4A, B. As in the previous simulations, we ran the simulation while varying the proportion of high-precision individuals, $p_H$. The posterior mean of the standard Bayesian correlation coefficient without correction increases as $p_H$ increases (light gray, panel B), while that of the corrected correlation (black) is closer to the true intersession correlation (0.71), regardless of $p_H$. For the corrected correlation, the 95% credible interval (dashed lines) narrows as $p_H$ increases, indicating decreasing uncertainty.

### Illustration with reinforcement learning model

For the reinforcement learning model, we use MAP estimates with SD($\alpha ^\prime$) = 1 in the application of Bayesian attenuation correction to the learning rates (examples of parameter estimates for $\alpha$ are shown in Supplementary Fig. S3). The results are shown in Fig. 4C, D. The correlation coefficient for the true values of the learning rate across sessions was approximately 0.76 (indicated by the horizontal dashed line in Fig. 4D). The correlation between the true parameters across sessions is not well estimated when there are no high-precision individuals ($p_H = 0$). However, it is well estimated when high-precision individuals are present, even if they are in the minority ($p_H = 0.2$), although the posterior mean tends to be smaller than the true value.

### Interim discussion

In this way, the Bayesian attenuation correction allows us to estimate the intersession correlation of the true parameter values, $\rho [\theta ^{(T1)}, \theta ^{(T2)}]$. This result can also be used to estimate the correlation between the true and estimated values, $\rho [\theta , \hat{\theta }]$. Let us suppose that the intersession correlation of the true parameters, $\rho [\theta ^{(T1)}, \theta ^{(T2)}]$, is are about 0.74, and the test–retest correlation between the estimates, $\rho [\hat{\theta }^{(T1)}, \hat{\theta }^{(T2)}]$, is about 0.3. From Eq. 7, we obtain $0.3 = 0.74 \cdot \rho [\theta , \hat{\theta }]^2$, so the correlation between the true parameter and its estimate is estimated to be $\rho [\theta , \hat{\theta }] = \sqrt{0.3/0.74} = 0.67$. In this scenario, the influence of the estimation error, characterized by a reduction factor of $0.67^2$, is greater than is the effect of the intersession variation, which has a reduction factor of 0.74. Therefore, we can conclude that in this example, to improve reliability, it is more effective to reduce the estimation error for each session, for example, by increasing the number of trials. However, it should be noted that there is uncertainty in the intersession correlation estimated by attenuation correction, test–retest correlation, and ICCs. Uncertainties are assessed by using credible intervals or confidence intervals. Researchers must interpret the results with these uncertainties in mind.

**Fig. 5:**
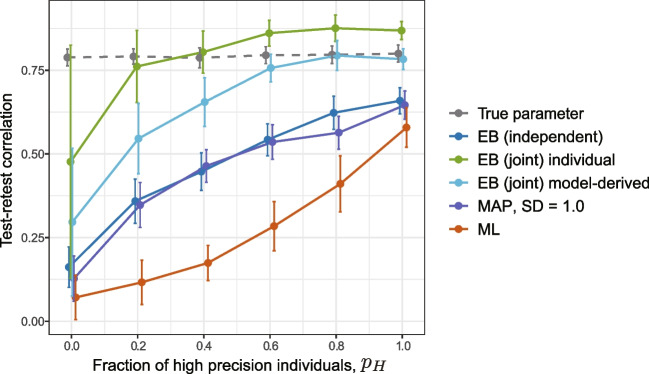
Evaluation of the reliability of parameters in a reinforcement learning model using EB joint models, when the true parameter varies across sessions (Case 2). The proportion of high precision individuals was varied in increments of 0.2 from 0 to 1. For each proportion, parameter estimation was conducted by using MAP estimation, ML estimation, EB (independent), and EB (joint). EB (independent) involves modeling (parameter estimation) independently for each session, whereas EB (joint) entails a joint modeling of the two sessions. For each condition and estimation method, 10 simulations were conducted, and the mean of the test–retest correlation was plotted. *Error bars* represent ±1 SD. For EB (joint), the test–retest correlation was calculated by using the MAP estimates of individual-level parameters as point estimates (EB (joint) individual) and by using the covariance matrix of the prior to calculate the correlation coefficients (EB (joint) model-derived). MAP, maximum a posteriori; ML, maximum likelihood; EB, empirical Bayes

### Inference of intersession correlation based on joint modeling

The method we have discussed thus far for estimating the intersession correlations of the true parameter employs a ‘two-step approach.’; Initially, parameter values and standard errors (SEs) are estimated for each individual. Then, hierarchical modeling is applied to the estimates to obtain the intersession correlation in a separate step. In previous studies on parameter reliability, a unified model integrating these processes has been employed (Brown et al., 2020; Waltmann et al., 2022; Mkrtchian et al., 2023; Sullivan-Toole et al., 2022; Yamamori, Robinson, & Roiser, 2023). Specifically, this approach involves joint estimation of the two sessions by using a hierarchical model, which incorporates a computational model at the individual level. The correlation between the corresponding parameters of the two sessions is then calculated from the estimated parameters of the group-level distribution (i.e., prior), akin to the method used in Bayesian attenuation correction. The correlation coefficient estimated in this way is called the *model-derived reliability* (e.g., Waltmann et al., 2022). Some researchers have interpreted correlations obtained through this method as a measure of parameter reliability (Brown et al., 2020; Waltmann et al., 2022; Mkrtchian et al., 2023; Sullivan-Toole et al., 2022; Yamamori, Robinson, & Roiser, 2023). The model-derived reliability is generally greater than is the test–retest correlation of the point estimates of parameters. However, importantly, this measure corresponds to $\rho [\theta ^{(T1)}, \theta ^{(T2)}]$, which captures only the intersession variability of the true parameter and does not incorporate the effect of estimation error. Therefore, model-derived reliability may measure only some of the factors that reduce reliability rather than improve it.

Figure 5 shows the results of such a joint model estimated for the simulation data with the same settings as the reinforcement learning model in Fig. 4D. Here, the EB approach estimated independently for each session is denoted as EB (independent), and the EB approach that models both sessions jointly is referred to as EB (joint) to distinguish between the two approaches. Model-derived intersession correlations with the EB (joint) are plotted as light blue lines. When the fraction of high-precision individuals, $p_H$, is greater than 0.6, this result is consistent with the intersession correlation of the true parameter values. Compared to the two-step approach (Fig. 4), the intersession correlation tends to be underestimated when $p_H$ is smaller than that, although the reason is unclear.

## Influence of the correlation of the estimation error for an individual

As previously mentioned, a key assumption of classical test theory is the independence of estimation errors between sessions. This assumption is likely valid when parameters for the two measures are estimated independently. However, caution is necessary when estimating the first and second sessions jointly, as discussed in the previous section. The covariance between the parameters of the group-level distribution (prior) can lead to correlated errors in the point estimates of individual-level parameters. For example, if an estimate is higher in the first session, it is likely to be higher in the second session as well, irrespective of the observed data. Consequently, this can lead to inflated correlations between estimates across sessions.

Let us confirm this with the results of the simulation shown in Fig. 5. The test–retest correlation for point estimates obtained by using EB (joint) (green line) seems to show a notable improvement compared to that obtained by using EB (independent) (blue line). However, this correlation exceeded the true intersession correlation of the true parameters. This result suggests that assuming covariance might break the independence between the two sessions, leading to inflated test–retest correlations. Consistent with this finding, Waltmann et al. (2022) also reported in their simulations that the use of point estimates by joint modeling leads to the overestimation of reliability.

## Recommendations for assessing and for reporting the reliability of the computational model parameters

In this paper, we have discussed the characteristics of test–retest reliability measures for parameter estimates in computational models. Reliability assessments are typically based on classical test theory; however, deviations from this theory are likely when using computational models. In particular, one key assumption of the theory, the uniformity of observation error, is often not met. We have also demonstrated that reliability is affected not only by errors in parameter estimation but also by intersession variations in the true parameters. Additionally, we investigated methods to assess these factors separately. In this section, we provide several recommendations and considerations for evaluating and reporting the reliability of computational model parameters based on these points.

### Which reliability measure should be used?

There are several types of reliability measures, including the ICC, Pearson’s correlation coefficient, and model-derived reliability measures, which are derived from the covariance matrix of a hierarchical model. Which reliability measures to focus on and how to use them depend on the purpose for which reliability is being measured.

The first point to consider is how to address intersession variation. If the goal is to develop a paradigm that captures the consistent behavioral tendencies of individuals by controlling for intersession variation, as in a typical experimental task indicator, ICC(A,1) may be appropriate because it also considers systematic variation and numerically indicates the presence of bias. However, there may be cases where the rank order relationships between individuals are important and systematic variation between sessions is not an issue. In such cases, the ICC (C,1) or Pearson’s correlation coefficient, which allows for systematic variation, may be an appropriate indicator. If the scale of variation of the estimates differs across sessions, ICC(C,1) and Pearson’s correlation coefficient take different values, and ICC(C,1) is lower because of the difference in scale. If the same priors are assumed for each session or if no priors are used (as in ML estimation), one would expect no noticeable change in the variance of the estimation error. In such a case, there is no significant difference between the ICC(C,1) and Pearson’s correlation coefficient. If the hierarchical model is used independently for each session, the variance in the prior may differ across sessions, and the amount of shrinkage may differ across sessions. In such a situation, the ICC(C,1) is lower than Pearson’s correlation coefficient. When the absolute value of the variance of a parameter is important (for example, in studies comparing the scale of the parameter with other studies), but systematic variation is not a problem, ICC(C,1) may be an appropriate measure.

When one is interested in the correlation coefficient with an external variable, the difference in scale due to shrinkage is not an issue, and the relative relationship of the parameter across individuals is often of interest. In such situations, it may make more sense to use Pearson’s correlation coefficient rather than ICC(C,1).

In other scenarios, one may wish to take advantage of intersession variability. For studies investigating within-individual variability, such as studies that use empirical sampling (e.g., Neuser et al., 2023), a lack of adequate random session-to-session variability may result in insufficient within-individual variability ($\sigma _\gamma ^2$). In such situations, it is more important to reduce per-session estimation error than intersession variation. Thus, evaluating the effects of intersession variation and estimation error separately may be beneficial in a framework such as the one proposed in this study. If the effect of estimation error is sufficiently small compared to intersession variation, then intersession variation may be desirable rather than problematic.

In studies reporting test–retest reliability of model parameters, it can be desirable to report as many of the measures considered in this paper as possible together. This may allow readers to use the necessary indicators for their purposes. In addition, because the direction of bias cannot be determined from ICC(A,1) alone (i.e., whether the parameter has become larger or smaller), it is desirable to report the mean of the estimates for each session, the coefficient of variation (e.g., Scheibehenne & Pachur, 2015), and test statistics (e.g., *t* value, *p* value, and confidence interval) for group differences (e.g., Mkrtchian et al., 2023; Toyama, Katahira, & Kunisato, 2023) so that the direction of bias can be assessed.

Some guidelines and reviews of reliability measures state that the Pearson’s correlation coefficient is not a suitable reliability measure (e.g., Koo & Li, 2016; Karvelis et al., 2023). However, as discussed in this paper, test–retest correlation can be a suitable reliability measure under certain conditions. Furthermore, for specific purposes, we argue that test–retest correlation may even be a more appropriate measure than ICCs.

### Parameter estimation methods and reporting of estimation results

The degree of heterogeneity in the estimation precision and the degree to which the prior affects reliability are also important pieces of information. First, it is desirable to provide reliability measures for both the estimation results obtained with ML with no prior and those obtained with MAP estimation with a fixed prior or EB to determine the extent to which the prior increases reliability metrics. Plotting the magnitude of estimation error (SE) is useful for determining whether heterogeneous groups are mixed, as shown in Fig. 2. The SE can be assessed through the Laplace approximation in ML or MAP estimations (Daw, 2011) or via the posterior distribution in Bayesian estimation. A future issue is to examine the method for visualizing precision and for determining heterogeneity.

Furthermore, when estimating the priors from data by using the EB or hierarchical Bayes method, there is a risk that the variance in the group-level distribution (prior) might become excessively small, leading to extreme shrinkage (Scheibehenne & Pachur, 2015; Sumiya & Katahira, 2020). In such a case, the reliability metric may reflect only some individuals with high precision; thus, it is advisable to evaluate the degree of shrinkage by plotting the point estimates or the prior (group-level distribution). This ensures that the variance of the prior is not too small compared to the range of possible parameters.

## Limitations and future directions

In this study, we introduced Bayesian attenuation correction as a method to estimate the intersession correlation of the true parameters. The validity of this method still requires further systematic simulations and analysis with real data. In addition, since this method assumes that the individual-level parameters are generated from a Gaussian distribution, the estimation results may be affected if the distribution of the true parameter deviates from the Gaussian distribution or if there are outliers. A robust correlation coefficient assuming a multivariate *t*-distribution has also been proposed for Bayesian correlation coefficients (Kruschke, 2013), and a method integrating it can be considered. When analyzing correlations with external variables, robust measures such as robust correlation coefficients or Spearman’s rank correlation coefficient can be employed. In such instances, reliability might be assessed by using a similar measure. Further study is needed on this point.

In this paper, we did not consider the intersession variation in the precision of each individual parameter, which may change over time or with mood or other conditions. Addressing what occurs in such scenarios and determining the suitable type of analysis for these cases remain open questions for future research.

While we considered the ideal situation where the model used for fitting was the true model, in reality, there is some divergence between the true computational process and the model. For example, in the context of reinforcement learning, we examined a standard model where the action value of the unselected option remains constant. However, models that postulate that the action value decreases toward a default value when not selected often agree better with empirical data (Ito & Doya, 2009; Katahira, Yuki, & Okanoya, 2017; Toyama, Katahira, & Ohira, 2019b). This finding suggests some degree of misspecification when analyzing real data using the standard reinforcement learning model. Even with model misspecification, parameter estimates can still be meaningful in characterizing the true process, as long as the model includes elements similar to the true process. For example, fitting the standard reinforcement learning model when the actual process is another type of reinforcement learning (e.g., actor-critic learning) still yields high correlations between the estimated and the true values for parameters such as learning rates (Katahira & Kimura, 2023). The impact of model misspecification on reliability is likely to vary depending on the true process and the model chosen. If the true process includes factors not accounted for in the model, individual differences in these factors may lead to unstable estimates. Conversely, the inclusion of redundant parameters may also destabilize and reduce the reliability of parameter estimates (Waltmann et al., 2022). Addressing these issues is an important focus of future research.

## Conclusion

In this paper, we reviewed the impact of deviations from classical test theory on the reliability of computational model parameters, as well as points to consider when interpreting the measures. We also discussed a framework to consider the influence of session-to-session variability and estimation error separately. Based on these insights, we offered recommendations for assessing and for reporting reliability. For modeling reward learning with reinforcement learning models, experimental tasks, models, and parameters are not consistent across studies, highlighting a lack of standardization in methods. To establish cognitive tasks and their modeling frameworks as reliable computational assays, it is crucial to develop guidelines so that the reliability of parameters can also be assessed by using standardized protocols.

## Open Practices Statement

All R scripts required to reproduce the results presented in this paper are available at https://osf.io/mqacz/. This paper does not contain the results of the experiment.

## Supplementary Information

Below is the link to the electronic supplementary material.

Supplementary file 1 (docx 1124 KB)

## Appendix

In this Appendix, for the sake of brevity in mathematical notation, session IDs are represented as ‘1’ and ‘2’ instead of ‘T1’ and ‘T2’.

## A The role of parameter reliability in correlation analysis

Here, we derive the relationship between the parameter reliability and the correlation coefficient between the parameter estimate $\hat{\theta }$ and the observed value $\hat{y}$ (e.g., self-reported symptom score) of the external variable *y* (e.g., true symptom severity). We assume that $\hat{y}$ is *y* plus the observation error and that the error is independent of the estimation error in the parameter estimates. Then, we have $\text {Cov}[\hat{y}, \hat{\theta }] = \text {Cov}[y, \theta ]$. From this relationship and the definition of Pearson’s correlation coefficient, we obtain

8 $$\begin{aligned} \rho [\hat{y}, \hat{\theta }]&= \frac{\text {Cov}[\hat{y}, \hat{\theta }]}{\sqrt{\text {Var}[\hat{y}]} \sqrt{\text {Var}[\hat{\theta }]}} \end{aligned}$$

9 $$\begin{aligned}&= \frac{\text {Cov}[y, \theta ]}{\sqrt{\text {Var}[\hat{y}]} \sqrt{\text {Var}[\hat{\theta }]}} \end{aligned}$$

10 $$\begin{aligned}&= \underbrace{ \frac{\text {Cov}[y, \theta ]}{\sqrt{\text {Var}[y]} \sqrt{\text {Var}[\theta ]}} }_{= \rho [y,\theta ]} \cdot \sqrt{ \frac{\text {Var}[y]}{\text {Var}[\hat{y}]}} \cdot \sqrt{\frac{\text {Var}[\theta ]}{\text {Var}[\hat{\theta }]}}. \end{aligned}$$

Here, $\text {Var}[y] / \text {Var}[\hat{y}]$ and $\text {Var}[\theta ] / \text {Var}[\hat{\theta }]$ represent the ratios of the variance of the true values *y* and $\theta$ to the variances of $\hat{y}$ and $\hat{\theta }$, respectively. According to Eq. 1, these ratios are the definitions of the reliability of each variable. From the above, we find the equation that expresses the relationship between the correlation coefficients between the parameter estimates and the external variables and the reliability of each variable:

11 $$\begin{aligned} \rho [\hat{y}, \hat{\theta }]&= \rho [y,\theta ] \cdot \sqrt{ \text {Reliability}(\hat{y}) \cdot \text {Reliability}(\hat{\theta }) }. \end{aligned}$$

The term ‘Reliability’ in the equation is often referred to as the ICC (Haines et al., 2023; Karvelis et al., 2023).

Let us consider the quantity $\text {Var}[\hat{\theta }]$ based on the schematic model presented in “Intersession variability of true parameters”. For the ICC(C,1) and test–retest correlation, where systematic intersession variation ($\eta$) is neglected, the variance is considered to be $\text {Var}[\hat{\theta }] = \sigma ^2_\theta + \sigma _\gamma ^2 + \sigma _\epsilon ^2$. In the case of ICC(A,1) because systematic variation is also taken into account, the variance is $\text {Var}[\hat{\theta }] = \sigma ^2_\theta + \sigma ^2_\eta + \sigma _\gamma ^2 + \sigma _\epsilon ^2$. Statistically, $\sigma _\gamma ^2$ and $\sigma _\epsilon ^2$ are indistinguishable, and both are treated as variances of the estimation error. If the sample of interest is a mixture of the session 1 (test) situation and the session 2 (retest) situation, the variance of $\hat{\theta }$ also includes the systematic between-session variance $\sigma ^2_\eta$ (for example, if individuals performing the task for the first time and individuals performing the task for the second time are included). In this case, the ICC(A,1) is a more appropriate measure of reliability. However, such a situation is rare, and it is more likely that in many cases, the relationship between $\hat{\theta }$ estimated from data obtained in a situation similar to the first session (a situation without practice effects) and external variables are examined. In such cases, the ICC(C,1) and test–retest correlation are appropriate measures.

## B Relationship between test–retest correlation and reliability

### B.1 Case where true parameters do not vary between sessions

First, we consider the case where parameters remain constant within an individual throughout the sessions. Let $\theta$ be a model parameter of interest. The population mean and variance of $\theta$ are denoted by $\mu _{\theta }$ and $\sigma _{\theta }^2$, respectively:

$$\begin{aligned} \text {E}[\theta ]&= \mu _{\theta }, \\ \text {Var}[\theta ]&= \sigma _{\theta }^2. \end{aligned}$$

Let $\theta _i$ be the true value of the parameter for the *i*-th individual and $\hat{\theta }_i$ be its estimate. We assume that the estimate for session *t* is generated by adding an estimation error $\epsilon _i$ to the true value:

$$\begin{aligned} \hat{\theta }_i^{(t)} = \theta _i + \epsilon _i^{(t)}. \end{aligned}$$

Here, we assume that the mean and variance of the errors are 0 (i.e., we assume unbiased estimators) and $\sigma _{\epsilon }^2$, respectively, for all individuals. Additionally, it is assumed that the errors are independent across different individuals and between the first and second sessions.

The population test–retest correlation (the value at which the Pearson’s correlation coefficient asymptotes in the limit of infinite sample size) can be expressed as

$$\begin{aligned} \rho [\hat{\theta }^{(1)}, \hat{\theta }^{(2)}]&= \frac{\text {Cov}[\hat{\theta }^{(1)}, \hat{\theta }^{(2)}]}{\sqrt{\text {Var}[\hat{\theta }^{(1)}]} \sqrt{\text {Var}[\hat{\theta }^{(2)}]}}, \end{aligned}$$

where $\cdot ^{(1)}$ and $\cdot ^{(2)}$ indicate the variables of the first and second sessions, respectively. By using the relationships

$$\begin{aligned} \text {Cov}[\hat{\theta }^{(1)}, \hat{\theta }^{(2)}]&= \text {E}[(\theta + \epsilon ^{(1)})(\theta + \epsilon ^{(2)})] - \text {E}[\theta + \epsilon ^{(1)}] \cdot \text {E}[\theta + \epsilon ^{(2)}] \\&= \text {E}[\theta ^2] - \text {E}[\theta ]^2 = \sigma _{\theta }^2, \end{aligned}$$

and

$$\begin{aligned} \sqrt{\text {Var}[\hat{\theta }^{(1)}]}&= \sqrt{\text {Var}[\hat{\theta }^{(2)}]} = \sqrt{\sigma _{\theta }^2 + \sigma _{\epsilon }^2}, \end{aligned}$$

we have

$$\begin{aligned} \rho [\hat{\theta }^{(1)}, \hat{\theta }^{(2)}]&= \frac{\sigma _{\theta }^2}{\sigma _{\theta }^2 + \sigma _{\epsilon }^2}. \end{aligned}$$

This result is consistent with the definition of reliability for cases without intersession variation in the true parameter.

### B.2 Case where the parameter varies between sessions

Next, we consider the more general case where the true parameters vary across sessions. The parameter estimate $\hat{\theta }_{i}^{(t)}$ for session *t* and individual *i* is assumed to be given by (see “Intersession variability of true parameters”):

$$\begin{aligned} \hat{\theta }_{i}^{(t)} = \bar{\theta }_i + \eta ^{(t)} + \gamma _{i}^{(t)} + \epsilon _i^{(t)}, \end{aligned}$$

where $\eta ^{(t)}$ is the systematic variation specific to session *t* and $\gamma _{i}^{(t)}$ represents the random variation in the true parameter for individual *i* in session *t*.

The covariance between the estimates in the first and second sessions is given by:

$$\begin{aligned} \text {Cov}[\hat{\theta }^{(1)}, \hat{\theta }^{(2)}]&= \text {E}[(\bar{\theta } + \eta ^{(1)} + \gamma ^{(1)} + \epsilon ^{(1)})(\bar{\theta } \!+\! \eta ^{(2)} + \gamma ^{(2)} \!+\! \epsilon ^{(2)})] \\&\quad - \text {E}[\bar{\theta } + \eta ^{(1)} + \gamma ^{(1)} + \epsilon ^{(1)}] \cdot \text {E}[\bar{\theta } + \eta ^{(2)} + \gamma ^{(2)} + \epsilon ^{(2)}] \\&= \text {E}[\bar{\theta }^2] - \text {E}[\bar{\theta }]^2 = \sigma _{\bar{\theta }}^2. \end{aligned}$$

The variance of the estimates for session *t* is

$$\begin{aligned} \text {Var}[\hat{\theta }^{(t)}]&= \text {E}[(\bar{\theta } + \eta ^{(t)} + \gamma ^{(t)} + \epsilon ^{(t)})^2] - \text {E}[\bar{\theta } + \eta ^{(t)} + \gamma _{i}^{(t)} + \epsilon ^{(t)}]^2 \\&= \sigma _{\bar{\theta }}^2 + \sigma _{\gamma }^2 + \sigma _{\epsilon }^2. \end{aligned}$$

Note that $\sigma _\eta ^2$ does not appear in this equation because $\eta ^{(t)}$ is an individual-independent constant for each session.

For this case, the population value of the test–retest correlation is

12 $$\begin{aligned} \rho [\hat{\theta }^{(1)}, \hat{\theta }^{(2)}]&= \frac{\text {Cov}[\hat{\theta }^{(1)}, \hat{\theta }^{(2)}]}{\sqrt{\text {Var}[\hat{\theta }^{(1)}]} \sqrt{\text {Var}[\hat{\theta }^{(2)}]}} = \frac{\sigma _{\bar{\theta }}^2}{\sigma _{\bar{\theta }}^2 + \sigma _{\gamma }^2 + \sigma _{\epsilon }^2}. \end{aligned}$$

Moreover, the intersession correlation between the true parameters is

13 $$\begin{aligned} \rho [\theta ^{(1)}, \theta ^{(2)}]&= \frac{\text {Cov}[\theta ^{(1)}, \theta ^{(2)}]}{\sqrt{\text {Var}[\theta ^{(1)}]} \sqrt{\text {Var}[\theta ^{(2)}]}} \nonumber \\&= \frac{\sigma _{\bar{\theta }}^2 }{\sigma _{\bar{\theta }}^2 + \sigma _{\gamma }^2}. \end{aligned}$$

From $\text {Cov}[\theta ^{(1)}, \hat{\theta }^{(1)}] = \sigma _{\bar{\theta }}^2 + \sigma _{\gamma }^2$, $\text {Var}[\theta ^{(1)}] = \sigma _{\bar{\theta }}^2 + \sigma _{\gamma }^2$, the correlations between the true parameter and the estimate in each session are

14 $$\begin{aligned} \rho [\theta ^{(1)}, \hat{\theta }^{(1)}] = \rho [\theta ^{(2)}, \hat{\theta }^{(2)}]&= \frac{\sigma _{\bar{\theta }}^2 + \sigma _{\gamma }^2}{\sqrt{\sigma _{\bar{\theta }}^2 + \sigma _{\gamma }^2} \sqrt{\sigma _{\bar{\theta }}^2 + \sigma _{\gamma }^2 + \sigma _{\epsilon }^2} } \nonumber \\&= \sqrt{\frac{\sigma _{\bar{\theta }}^2 + \sigma _{\gamma }^2}{\sigma _{\bar{\theta }}^2 + \sigma _{\gamma }^2 + \sigma _{\epsilon }^2}} \end{aligned}$$

Therefore, the following equation holds for these correlation coefficients:

15 $$\begin{aligned} \rho [\hat{\theta }^{(1)}, \hat{\theta }^{(2)}] = \rho [\theta ^{(1)}, \theta ^{(2)}] \cdot \rho [\theta ^{(1)},\hat{\theta }^{(1)}] \cdot \rho [\theta ^{(2)}, \hat{\theta }^{(2)}]. \end{aligned}$$

We can confirm that this relationship holds by substituting (12), (13) and (14) into this equation. This equation corresponds to Eq. 7 in the main test. This reveals that the test–retest correlation of parameter estimates can be decomposed into two distinct parts: the intersession correlation of the true parameter and the correlation between the true parameter and the estimate for each session.

## C A Gaussian response model and estimation methods

To illustrate the properties of test–retest correlation, we use a one-dimensional Gaussian response model (Gelman et al., 2013; Katahira, 2016). The model parameter at the individual (subject) level is the mean parameter $\theta$, which can vary between individuals. Let us suppose there are *N* individuals, and we denote the true value of $\theta$ for the *i*th individual by $\theta _i$. Here, we consider the case where $\theta$ is constant throughout the session. In this case, $\theta$ is assumed to be drawn from a common Gaussian distribution with mean $\mu$ and variance $\sigma _{\theta }^2$:

16 $$\begin{aligned} \theta _i \sim \mathcal {N}(\mu , \sigma _{\theta }^2). \end{aligned}$$

The response $x_{it}$ of the *i*-th individual at the *t*-th trial is assumed to follow a one-dimensional Gaussian distribution:

17 $$\begin{aligned} x_{it} \sim \mathcal {N}(\theta _i, \nu _i^2). \end{aligned}$$

Here, $\nu _i^2$ represents the variance of the response at each trial for the *i*-th individual. The ML estimator for $\theta _i$ is the sample mean, which follows a Gaussian distribution:

18 $$\begin{aligned} \hat{\theta }_i^{\text {(ML)}} = \bar{x}_i = \frac{1}{T_i} \sum _{t=1}^{T_i} x_{it} \sim \mathcal {N}(\theta _i, \sigma _i^2), \end{aligned}$$

where $\sigma _i = \frac{\nu _i}{\sqrt{T_i}}$, which corresponds to the SE of this estimator (i.e., $\sigma _\epsilon$ in the notation of the main text) and $T_i$ denotes the number of trials for individual *i*. We assume that the individuals in a proportion $p_H$ of the entire population have a low $\sigma _i$ (i.e., high-precision individuals; $\sigma _i = \sigma _H$), while the remaining individuals in proportion $1 - p_H$ have a high $\sigma _i$ (i.e., low-precision individuals; $\sigma _i = \sigma _L$).

We assume that the prior distribution of $\theta _i$ is a Gaussian distribution with mean $\lambda$ and SD $\tau$. Then, the posterior distribution of $\theta _i$ is also Gaussian, with mean $(1 - w_i) \lambda + w_i \bar{x}_i$ and variance $\frac{1}{1/\tau ^2 + 1/\sigma _i^2}$, with a weight parameter:

19 $$\begin{aligned} w_i = \frac{1/\sigma _i^2}{1/\tau ^2 + 1/\sigma _i^2}. \end{aligned}$$

The MAP estimate for $\theta _i$ is the posterior mean (i.e., $\hat{\theta }_i^{\text {(MAP)}} = (1 - w_i) \lambda + w_i \bar{x}_i$).

For the independent ML estimation, the true test–retest correlation coefficient is given by

20 $$\begin{aligned} \rho [\hat{\theta }^{\text {(ML, 1)}}, \hat{\theta }^{\text {(ML, 2)}}] = \frac{\sigma _\theta ^2}{\sigma _\theta ^2 + p_H\sigma _H^2 + (1 - p_H) \sigma _L^2}. \end{aligned}$$

For the MAP estimation, the true test–retest correlation coefficient given the prior SD, $\tau$, is

21 $$\begin{aligned} \rho [\hat{\theta }^{\text {(MAP,1)}}, \hat{\theta }^{\text {(MAP,2)}})]&= \frac{\sigma _\theta ^2}{\sigma _\theta ^2 + \frac{p_H w_H^2 \sigma _H^2 + (1 - p_H) w_L^2 \sigma _L^2}{p_H w_H^2 + (1 - p_H) w_L^2}}, \end{aligned}$$

with

22 $$\begin{aligned} w_L = \frac{1/\sigma _L^2}{1/\tau ^2 + 1/\sigma _L^2} \text { and } w_H = \frac{1/\sigma _H^2}{1/\tau ^2 + 1/\sigma _H^2}. \end{aligned}$$

These equations imply that the retest correlation of this model does not depend on the mean of the prior $\lambda$.

From Eq. 21, we find that the variance of the estimation error ($\sigma _\epsilon ^2$) in Eq. 2 for test–retest correlations in the case of MAP estimation corresponds to a weighted average of the estimation variance given by

$$\begin{aligned} \frac{p_H w_H^2 \sigma _H^2 + (1-p_H)w_L^2 \sigma _L^2}{p_H w_H^2 + (1-p_H)w_L^2}. \end{aligned}$$

Here, $w_L$ and $w_H$ are the weights for the low-precision individuals and the high-precision individuals, respectively. This weight depends on the variance of the prior distribution (denoted by $\tau$) and the variance of the estimation error, as in Eq. 22. Therefore, the results of MAP estimation depend on the SD of the prior.

The EB method provides population parameter estimates ($\lambda , \tau$) to maximize the marginal likelihood. The estimates for the population parameters satisfy the following two equations:

23 $$\begin{aligned} \lambda&= \frac{\sum _{i=1}^N \frac{1}{\sigma _i^2 + \tau ^2} \bar{x}_i}{\sum _{i=1}^N \frac{1}{\sigma _i^2 + \tau ^2}}, \end{aligned}$$

24 $$\begin{aligned} \sum _{i=1}^N \frac{1}{\sigma _i^2 + \tau }&= \sum _{i=1}^N \frac{(\bar{x}_i - \lambda )^2}{(\sigma _i^2 + \tau )^2}. \end{aligned}$$

For details of the derivation, see Appendix C in Katahira (2016). The population parameter estimates ($\lambda , \tau$) can be obtained by solving these equations.

## D Simulation settings

The settings for the simulations are as follows. All the simulations, statistical analyses, and plots were performed by using R version 4.2.2 (R Core Team, 2015).

### D.1 Gaussian response model

The simulation setup for the Gaussian response model is as follows. For each simulation, data were generated for 200 individuals based on the Gaussian response model. The baseline $\bar{\theta }$ for each individual’s parameter was generated from a Gaussian distribution with a mean of 1 and an SD of 1. In the examples in Fig. 1C and D, the fraction of high-precision individuals, $p_H$, was set to 0.5. In this example, the baseline parameter $\bar{\theta }$ was used as the true parameter $\theta$ for each session. In the simulations in Fig. 4A and B, the parameters for each session were generated by adding a Gaussian random number with a mean of 0 and SD = $\sqrt{0.4}$ to $\bar{\theta }$. This gives the correlation between $\theta ^{(1)}$ and $\theta ^{(2)}$ of $1/(1 + 0.4) = 0.71$. For both cases, the SD of the response of the high-precision individual was set to 0.5 and that of the low-precision individual was set to 2.0. The number of trials was set to 1 for all individuals. The ML estimate in this case is the observed value of each individual itself. For the EB, the mean and the variance of the Gaussian prior were optimized by iteratively computing Eqs. 23 and 24.

### D.2 Reinforcement learning model

We simulated the choice behavior on a probabilistic reversal learning task consisting of 100 trials by using standard reinforcement learning models (described in the main text). For each simulation run, we assumed 200 virtual participants, with each participant’s behavior being generated from the reinforcement learning model with unique parameters for each individual. Of the two options, one option was associated with a high reward probability of 0.8. The other option was associated with a low reward probability of 0.2. Rewards were given based on the reward probability assigned to the chosen option. After 50 trials, the reward probabilities of the two options were flipped.

The true parameters used in the simulation were generated as follows. Let us denote the learning rate and inverse temperature for the *i*-th participant $(i=1,\ldots ,200)$ in session *t* $(t = 1,2)$, as $\alpha _i^{(t)}$ and $\beta _i^{(t)}$, respectively. We used two auxiliary parameters to maintain consistency with the EB method (Huys, Moutoussis, & Williams, 2011; Huys et al., 2012; Waltmann et al., 2022): $\alpha _i^{\prime (t)}$, a logit parameter that is transformed into the learning rate through logit transformation, and $\beta _i^{\prime (t)}$ is the logarithm of the original inverse temperature and is thus called the log parameter. These auxiliary parameters were subsequently transformed into the original model parameters as follows:

25 $$\begin{aligned} \alpha _i^{(t)}&= \frac{1}{1 + \exp (-\alpha _i^{\prime (t)})}, \end{aligned}$$

26 $$\begin{aligned} \beta _i^{(t)}&= \exp (\beta _i^{\prime (t)}). \end{aligned}$$

The auxiliary parameters were generated as follows. First, individuals were divided into two groups: a low-precision group and high-precision group. For Case 1 (Fig. 2), the parameters did not change between sessions; namely, $\alpha _i^{(1)} = \alpha _i^{(2)}$, $\beta _i^{(1)} = \beta _i^{(2)}$. In Case 1, $\alpha _i^{\prime (t)}$ was assumed to follow a Gaussian distribution with a mean of -1 and a SD of 1, regardless of the group. The log parameter $\beta _i^{\prime (t)}$, for the low-precision group, was generated from a Gaussian distribution with a mean $\mu _{\beta ^\prime ,L} = -0.5$, and an SD $\sigma _{\beta ^\prime } = 0.2$; for the high-precision group, it was generated from a Gaussian distribution with a mean $\mu _{\beta ^\prime ,H} = 1.4$, and an SD $\sigma _{\beta ^\prime } = 0.2$.

For the simulation in Case 2 (Figs. 4 and 5), we assumed that the parameters varied between sessions. Specifically, the auxiliary parameters for two sessions were jointly generated from a bivariate Gaussian distribution. The logits of the learning rate, $[\alpha _i^{\prime (1)},\alpha _i^{\prime (2)}]$, were generated from a bivariate Gaussian distribution with a mean vector of [-1, -1], a covariance matrix whose diagonal components are $\sigma _{\alpha ^\prime }^2 = 1^2 = 1$, and off-diagonal components are $0.8 \times \sigma _{\alpha ^\prime }^2=0.8$. This yields an intersession correlation of 0.79 for the transformed learning rate. For the log parameters of the inverse temperature $[\beta _i^{\prime (1)},\beta _i^{\prime (2)}]$, the mean vector was $[-0.5, -0.5]$ for low-precision individuals, [1.4, 1.4] for high-precision individuals, diagonal components of the covariance matrix are $\sigma _{\beta ^\prime }^2 = 0.2$ and off-diagonal components are $0.8 \times \sigma _{\beta ^\prime }^2 = 0.032$ for both groups.

For both cases, the proportion of high-precision individuals, $p_H$, was varied from 0 to 1 in increments of 0.2. In Case 1, three estimation methods (MAP, ML, and EB (independent)) were employed for each dataset, with each condition being simulated 50 times. However, for EBs, due to computational time constraints, estimations were performed only for the first ten simulations. In Case 2 (Figs. 4 and 5), each condition was simulated 50 times (with 20 simulations for the EB methods), and the estimations were conducted by using the EB (independent), EB (joint), MAP, and ML.

The MAP estimation with a fixed prior and ML determine parameters to maximize the probability (likelihood) that the model produces a given choice of data (in ML) or the posterior probability of multiplying this likelihood by a prior probability (in MAP). For the reinforcement learning models, maximization was performed by using the rsolnp package (version 1.15), which implements the augmented Lagrange multiplier method with an SQP interior algorithm. To avoid being trapped by trivial local solutions, the algorithm was initialized five times with random starting values, and the parameter set giving the smallest negative log-likelihood (in ML) or log-posterior (in MAP) was chosen.

In the MAP estimation with a fixed prior, a Gaussian distribution with a mean of 0 was assumed as the prior for the logit parameter $\alpha ^{\prime }$ of the learning rate. Note that this mean is different from the mean of the generative distribution of $\alpha ^{\prime }$, which is -1. For this prior, three different variance values were considered: 0.5, 1.0, and 1.5. MAP estimation was conducted separately for each of these variance assumptions.

For the log parameter $\beta ^\prime$ for the inverse temperature, we set up a Gaussian prior distribution with the same mean and variance as the distribution of the true parameter to reduce the impact of mismatch between the prior and the true parameter distribution. The mean and variance of the true parameter distribution for the entire population depend on the proportion of high-precision individuals, $p_H$. Specifically, the mean of this distribution is given by $p_H \cdot \mu _{\beta ^\prime ,H} + (1-p_H) \cdot \mu _{\beta ^\prime ,L}$, and its variance is given by $p_H (1 - p_H) (\mu _{\beta ^\prime ,L} - \mu _{\beta ^\prime ,H})^2 + \sigma _{\beta ^\prime }^2$.

In the application of EB to the reinforcement learning model, the EM algorithm was used to optimize the individual-level parameters and the group-level distribution parameters that served as the prior distribution (Huys et al., 2012; Huys, Pizzagalli, Bogdan, & Dayan, 2013). The algorithm alternates between estimating the individual level parameters (E-step) and updating the group level (prior) parameters (M-step) to maximize the marginal likelihood. The computation was terminated when the criterion that the average change in the log-posterior likelihood of the mean per individual was less than 0.001 was met. Due to the limitation of computation time, the maximum number of iterations was set to 50, and the computation was terminated at 50 iterations even if the criterion was not met.

In EB (independent), parameters for each session are estimated separately, assuming a multivariate Gaussian prior distribution with a covariance matrix for the prior of the auxiliary parameters, $\alpha ^{\prime }$ and $\beta ^{\prime }$.

For the EB (joint), which estimates two sessions simultaneously, a Gaussian prior (with covariance) for four parameters, $\alpha ^{\prime (1)}$, $\alpha ^{\prime (2)}$, $\beta ^{\prime (1)}$, and $\beta ^{\prime (2)}$ is assumed. In the EB (joint), the Pearson’s correlation coefficients of the parameters between the two sessions can be derived from the covariance matrix of the prior. That is, the correlation coefficient is obtained by dividing its covariance by the sum of the square roots of the variance in each session (Waltmann et al., 2022). Figure 5 plots the intersession correlation obtained in this way, but it is the intersession correlation of the logit $\alpha ^\prime$ rather than the intersession correlation of the learning rate $\alpha$. However, as in the results reported in Appendix D.2, the impact of the difference is likely to be small.

### D.3 Bayesian attenuation correction

We consider Bayesian attenuation correction (Behseta et al., 2009; Matzke et al., 2017) as a method for estimating the intersession variability of true parameters. This method treats parameter uncertainty as known data. Specifically, we define a generative model as follows: The true parameter values (denoted as $\theta _1$ for the first session and $\theta _2$ for the second session) are assumed to be generated from a multivariate Gaussian distribution (group-level distribution):

27 $$\begin{aligned} \begin{bmatrix} \theta _1 \\ \theta _2 \end{bmatrix} \sim \mathcal {N} \left( \begin{bmatrix} \mu _1 \\ \mu _2 \end{bmatrix}, \begin{bmatrix} \sigma _1^2 &{} \rho \sigma _1 \sigma _2 \\ \rho \sigma _1 \sigma _2 &{} \sigma _2^2 \end{bmatrix} \right) . \end{aligned}$$

Here, $\mu _1$ and $\mu _2$ are the mean parameters, and $\sigma _1$ and $\sigma _2$ are the SDs of $\theta _1$ and $\theta _2$, respectively. $\rho$ represents the correlation coefficient between $\theta _1$ and $\theta _2$. Gaussian random variables with given SDs (SEs) are assumed to be added to each true value to obtain observed values (in our context, parameter estimates).

The posterior distributions of the parameters in this hierarchical generative model are obtained via Bayesian estimation. The posterior of $\rho$ is interpreted as the posterior of the intersession correlation of the true parameters across sessions. The prior for each mean parameter $\mu _1, \mu _2$ follows a Gaussian distribution with a mean of 0 and a precision of 0.001, implying a variance of 1000. We assume a uniform distribution of [0, 100] for the prior of the SD parameters, $\sigma _1$, $\sigma _2$. For the correlation coefficient $\rho$, a uniform prior distribution of [-1, 1] is assumed. Assuming no estimation error of individual parameters, i.e., setting the SE of all individual parameters to zero, this method yields a Bayesian solution of the standard Pearson’s correlation coefficient (Ly, Marsman, & Wagenmakers, 2018). In this paper, the coefficient obtained in this manner is referred to as the standard Bayesian correlation coefficient.

To estimate the posterior distribution of parameters of this generative model, we used Stan 2.26.1 with the interface RStan v2.32.3. Stan employs a Hamiltonian Monte Carlo (HMC) approach to sample from the posterior distribution. A total of 4000 samples were drawn after 1000 burn-in samples for each of the three chains. Therefore, the total sample size was 12000. The Gelman–Rubin $\hat{R}$ statistic (Gelman & Rubin, 1992) was monitored to confirm that the MCMC chains converged to the target distributions ($\hat{R}$ values close to 1.00 imply convergence). A $\hat{R}$ statistic less than 1.05 indicates good convergence (Gelman et al., 2013). For all the cases for the Gaussian response model, $\hat{R}$ was less than 1.05. indicating satisfactory convergence. For the reinforcement learning model, $\hat{R}$ was less than 1.05 except for the Bayesian attenuation correction when $p_H$ was 0 (consisting only of low-precision individuals), where $\hat{R}$ was approximately 1.36.

For the Gaussian response model, the SE values were analytically obtained (i.e., $\frac{1}{1/\tau ^2 + 1/\sigma _i^2}$) for each parameter In the reinforcement learning example in this study, the SE was computed by using the Laplace approximation based on the MAP estimates. The Laplace approximation uses the numerically obtained second derivative of the log posterior probability function of the parameter estimates, which are summarized as a Hessian matrix (Daw, 2011). The SE is then approximated as the root of the corresponding diagonal elements of the inverse of the Hessian matrix. Furthermore, in the reinforcement learning model considered in this study, the original parameters are transformed versions of these estimates. Thus, further approximation is required to obtain the SE of the original parameters. For the learning rate $\alpha$, the SE is approximated by $[\text {logit}(\hat{\alpha }^\prime + \text {SE}(\hat{\alpha }^\prime )) - \text {logit}(\hat{\alpha }^\prime - \text {SE}(\hat{\alpha }^\prime ))]/2$, where $\text {SE}(\hat{\alpha }^\prime )$ indicates the SE obtained as above. Note that this is a coarse approximation, particularly in regions where the transformation is highly nonlinear.

## Footnotes

1 In this paper, we focus on point estimates when discussing parameter estimation. In the context of Bayesian estimation, where parameter uncertainty is assessed through a posterior distribution, the term ‘estimation error’ may not be appropriate.

2 The term ‘true parameters’ is more accurate if they are considered values to which the estimate converges when the number of trials is infinite. When the fitted model is incorrect, the estimated parameters are biased and do not align with those of the true generative process (Nassar & Gold, 2013; Katahira, 2018; Toyama, Katahira, & Ohira, 2019a). Relatedly, we assume the mean of the estimation error to be zero. However, in real-world scenarios, there might be a bias that causes this mean to deviate from zero. As reliability is a measure concerning the deviation of parameter estimates from the mean, rather than from their true values, such bias can be appropriately disregarded in discussions about reliability.

3 Even when $p_H = 0$ or $p_H = 1$, the estimation precision within groups is not uniform due to differences in parameters and stochastic choice patterns, so there are subtle differences between estimation methods.

4 In Hedge, Powell, and Sumner (2018), the ICC is defined as $\text {ICC} = \frac{\text {Variance between individuals}}{\text {Variance between individuals} + \text {Error variance} + \text {Variance between sessions}}$. Here, the variance between sessions refers to the variance due to systematic changes (bias), i.e., $\sigma _{\eta }^2$.

5 Although it is possible to treat the session $\times$ individual interaction as a mixed effect model, in the case of model parameter estimation, a single estimate is derived for each session, and since it is not a repeated measure, such interaction cannot be distinguished from error.
