# Supplementary material for: Does the reliability of computational models truly improve with hierarchical modeling? Some recommendations and considerations for the assessment of model parameter reliability: Reliability of computational model parameters
Source: Psychon Bull Rev. 2024 May 8;31(6):2465–86. doi: 10.3758/s13423-024-02490-8 (PMC11680638; doi:10.3758/s13423-024-02490-8)
Supplement: Supplementary file 1 — (docx 1124 KB) [file 13423_2024_2490_MOESM1_ESM.docx]

**Supplementary materials**


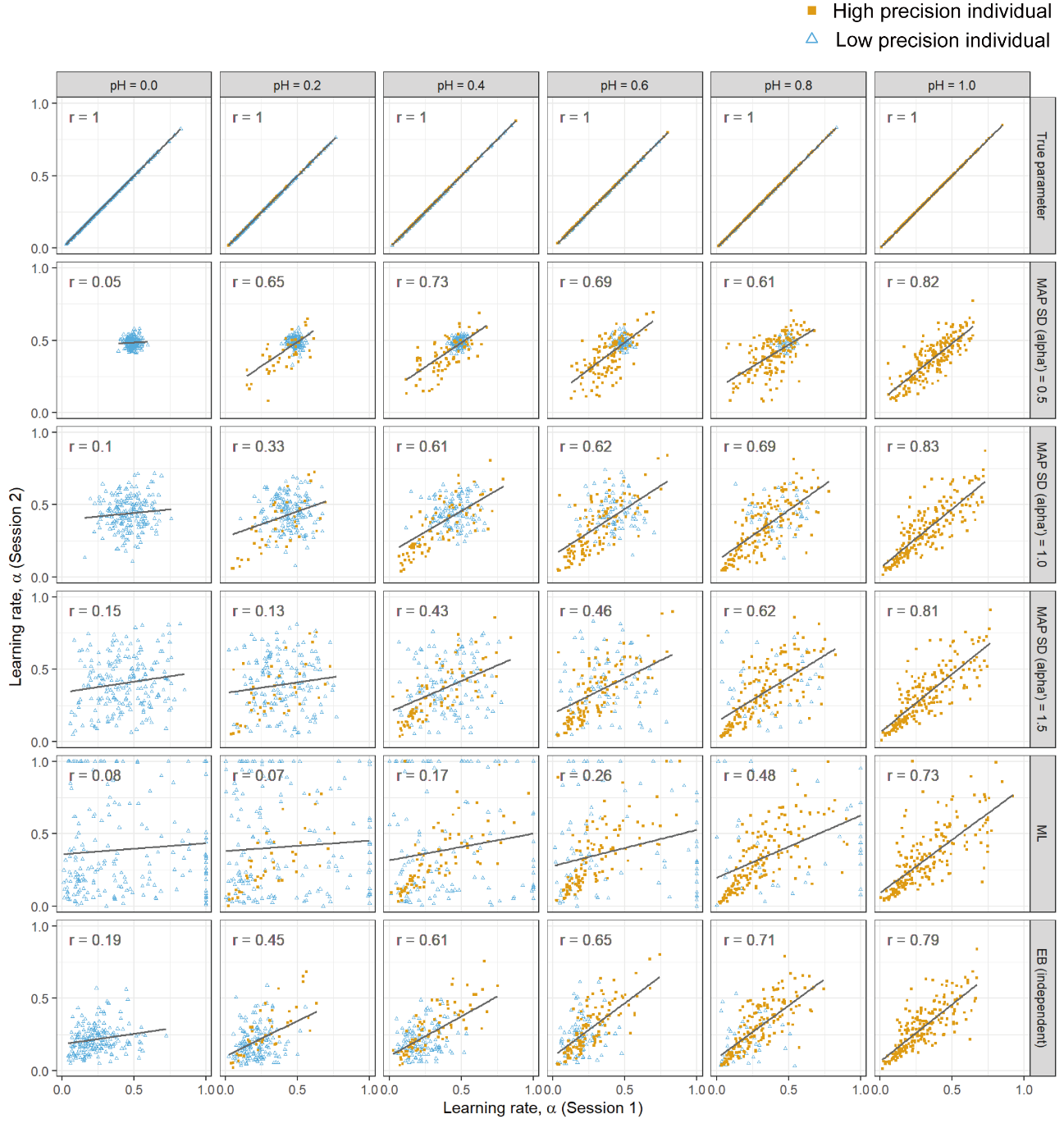


**Fig. S1　Scatterplot of the parameter estimates for learning rate, α, in the case with no intersession variability in the true parameter.** Orange dots represent high-precision individuals, and light blue dots represent low-precision individuals. The solid line represents the regression coefficient across all populations. r, Pearson correlation coefficient; MAP, maximum a posteriori; ML, maximum likelihood; EB, empirical Bayes.


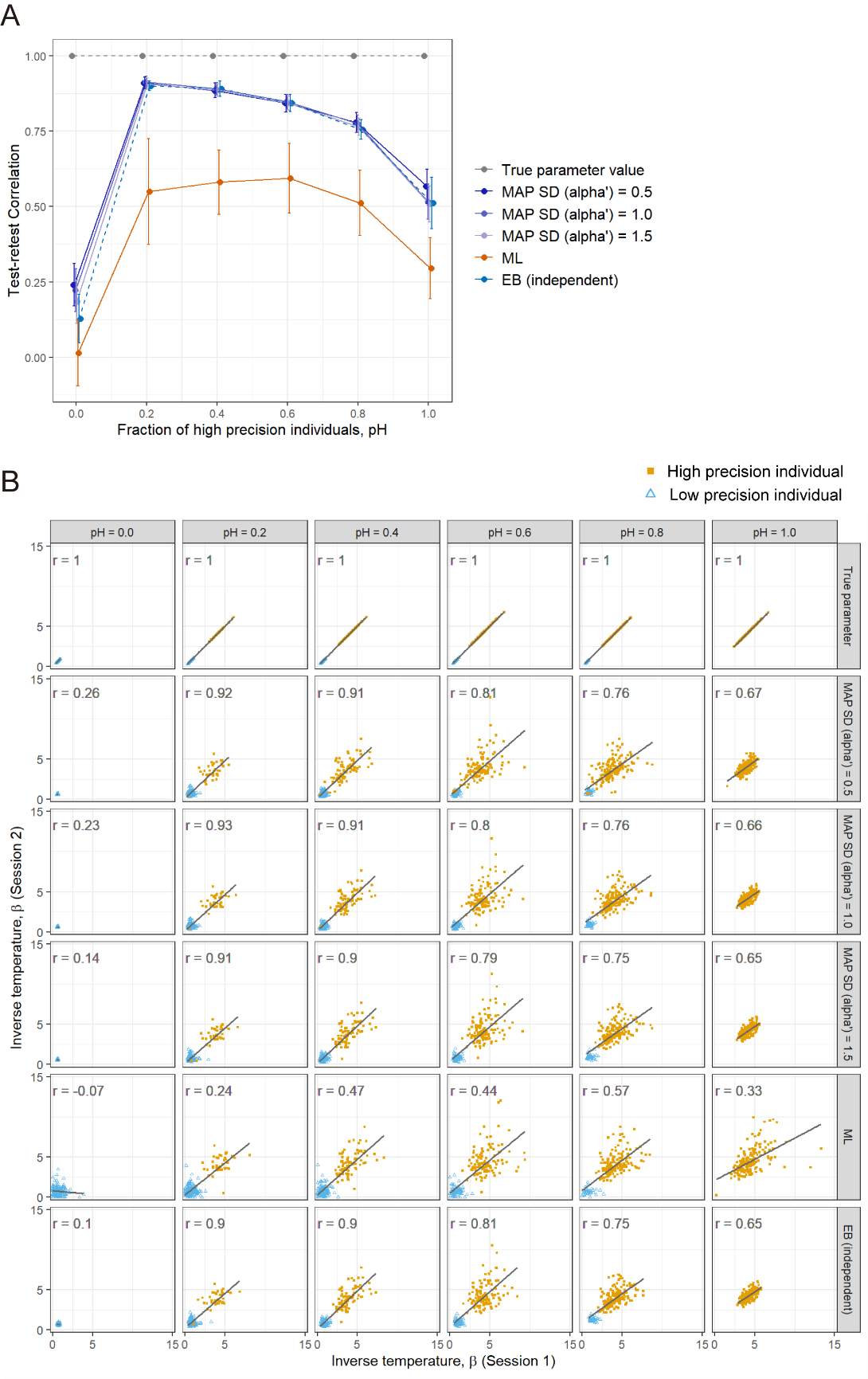


Fig. S2　Results for inverse temperature, $\beta$, in reinforcement learning in the case with no intersession variability in the true parameter. A: Test-retest correlation of estimates for $\beta$. B. Scatterplot of the estimates for $\beta$. The conventions are the same as those for Fig. S1 and Fig. 2 of the main text.


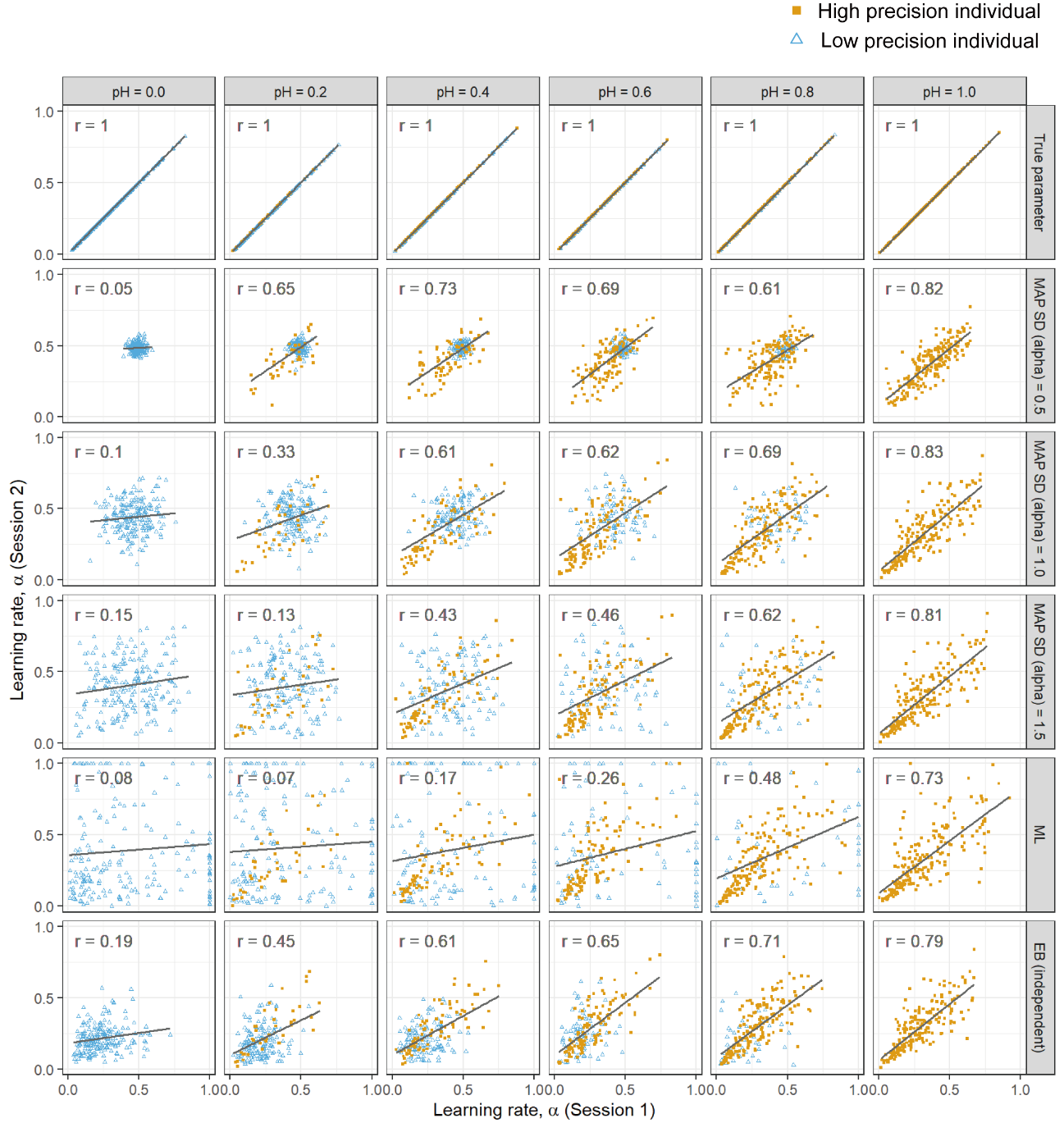


Fig. S3　Scatterplot of the estimated values of the parameter α in the case with intersession variability of the true parameter. The convention is the same as that for Fig. S1. The convention is the same as that for Fig. S1.
